# Impact of Environmental Moral Education on Pro-Environmental Behaviour: Do Psychological Empowerment and Islamic Religiosity Matter?

**DOI:** 10.3390/ijerph18041604

**Published:** 2021-02-08

**Authors:** Abida Begum, Liu Jingwei, Maqsood Haider, Muhammad Maroof Ajmal, Salim Khan, Heesup Han

**Affiliations:** 1 School of Marxism, Northeast Forestry University, Harbin 150040, China; abidakhg@gmail.com; 2 Department of Management Sciences, FATA University, FR Kohat 25000, Pakistan; dr.haider@fu.edu.pk; 3 University Institute of Management Sciences, PMAS-Arid Agriculture University, Rawalpindi 46000, Pakistan; dr.maroof@uaar.edu.pk; 4 School of Management, Harbin Institute of Technology, Harbin 150001, China; salimuom@yahoo.com; 5 College of Hospitality and Tourism Management, Sejong University, Seoul 05006, Korea; heesup@sejong.ac.kr

**Keywords:** environmental moral education, psychological empowerment, Islamic religiosity, pro-environmental behaviour, Pakistani university students

## Abstract

In light of increasing concerns about global environmental problems, environmental moral education is assumed to have a significant influence on the pro-environmental behaviour of students. Within the past decade, several higher education institutes have acknowledged the importance of integrating sustainability into the educational curriculum to have a focused and explicit impact on society. The current study investigated the relationship between environmental moral education and pro-environmental behaviour while drawing upon insights from the conservation of resource theory. The relationship among the aforementioned variables was studied for the mediating role of psychological empowerment and the moderating effect of Islamic religiosity. Data were collected from 429 university students with a cross-sectional approach. The data were analysed using “structural equation modelling” and “PROCESS” analytical techniques. The results of the study followed the predicted conceptual model, that is, environmental moral education was positively related to pro-environmental behaviour. Furthermore, psychological empowerment partially mediated the aforementioned relationship, while Islamic religiosity moderated the relationships between environmental moral education and pro-environmental behaviour as well as between environmental moral education and psychological empowerment. These findings reinforce the importance of environmental moral education and Islamic religiosity in understanding the Muslim student’s ecological behaviours.

## 1. Introduction

Environmental education is an essential component of biodiversity preservation interventions. Environmental education provides awareness and sensitivity about environmental problems, increases knowledge and helps in attaining positive attitudes toward environmental threats [1,2]. Education plays a vital role in explaining high-level environmental behaviours and concerns.

Highly educated (those perusing or who graduated from bachelor’s degree programs are considered as educated persons, whereas those enrolled or graduated from master’s or higher degree programs are considered as highly educated persons) persons are more motivated to bettering the environment, because education produces awareness about the potential damage to the environment [3,4]. Mostly, education provides a high level of knowledge and awareness about environmental problems and its solutions which results in sustainable and pro-environmental behaviour (PEB) [5,6]. It is believed that, currently, human behaviour has detrimental impacts on the environment, and, especially, younger generations will be affected more owing to the current global environmental problems which will continue to become worse in the future if not well addressed [6,7]. Therefore, it is the necessity of time to understand and improve the ecological behaviours of individuals. Higher education is increasing the responsibility of individuals, providing the ecological education, skills and ethics required for a sustainable and improved world. Therefore, universities have a pivotal role in obliging pro-environmental behaviour; they also play a vital role in transforming societies toward environmental sustainability [8,9]. Recently, universities began promoting pro-environmental and sustainable development through education, research, incorporating sustainable development into the institutional agenda and encouraging various programs on training, awareness and development of staff for PEB. A comprehensive review of more than a dozen research papers on the effect of education on PEB showed that education may make individuals aware of their PEB [10]. The leaders of tomorrow are being trained in universities; therefore, it is imperative to provide them with environmental education and ethics so they became psychologically empowered and their attitude changes toward pro-environmental behaviours, which could turn society toward environmental sustainability [4,11,12].

**Figure 1:**
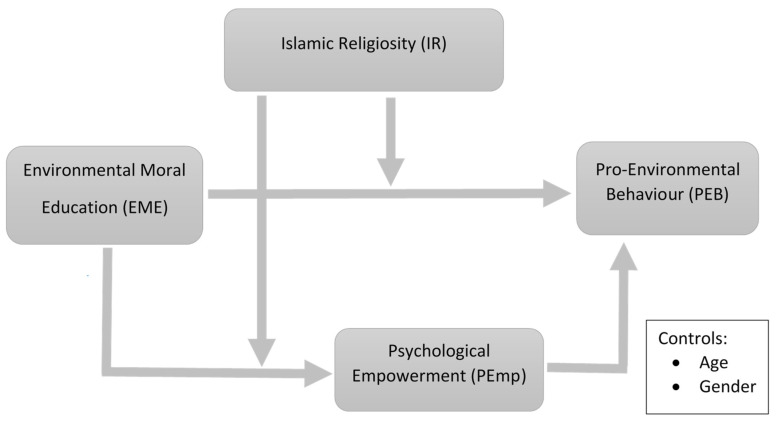
Theoretical framework of the study.

The understanding of one’s predisposition to adopt PEB is a complex issue that is still not fully understood. In the past, various factors have been considered that affect PEB such as personal norms, attitudes, intentions, value orientations, environmental concerns, self-identity, etc. [11,12,13,14,15,16,17]. Psychological empowerment can also play an essential role in improving ecological behaviours, as it empowers an individual with the feeling of “the power to change things” and motivates consumers to preserve the environment [18]. Hence, the current study proposes that environmental moral education is directly and indirectly (via psychological empowerment) positively related to pro-environmental behaviour. Besides, given the role of Islamic teachings regarding environmental protection, such as balancing of the environment, environmental consciousness, conservation of resources, such as water, trees, etc. [19], the current study proposes Islamic religiosity as a boundary condition to the prior proposed relationships (see Figure 1 for the conceptual framework of the study).

This study makes two significant contributions to the literature. First, the study extends the range of psychological processes to mediate the relationship between environmental moral education and pro-environmental behaviour. Until now, very few psychological processes (e.g., affect and attitudes) have been considered as the mediator between environmental moral education and pro-environmental behaviour [5,20]. This study considers psychological empowerment as a mediator to explain the relationship between environmental moral education and pro-environmental behaviour. Second, despite the growing importance of Islamic religiosity in environmental literature, its role in pro-environmental behaviour is very limited [21]. By proposing Islamic religiosity as the moderator between environmental moral education and pro-environmental behaviour, this study extends its scope to pro-environmental literature. Hence, the objective of this study was to gain a better understanding of the relationship between environmental moral education and pro-environmental behaviour by exploring the mediating role of psychological environment. The study also aimed to extend the role of Islamic religiosity in pro-environmental behaviour.

In the succeeding section, first, the conceptual model along with the hypotheses of the study are discussed. Next, the methodology of the study and the results and analysis are presented. The hypotheses are tested by structural equation modelling using AMOS (Singapore) and SPSS (Chicago, IL, USA) software. Data were collected from undergraduate and postgraduate students. Finally, discussions, conclusions and future recommendations are given.

## 2. Literature Review

### 2.1. Environmental Moral Education and Pro-Environmental Behaviour

Education about, from and for the environment is called environmental education or environmental moral education (EME) [22]. Knowledge and understanding of the environment are developed from EME, which further offers potential skills benefiting the environment. Education from the environment can be achieved using the outdoors as a learning resource to enhance learning, whereas a sense of awareness and responsibility is developed for the environment through EME, which positively affects the attitudes and behaviours toward a green ecological lifestyle [23]. Environmental moral education is considered an essential component of biodiversity conservation interventions [2]. The increase in knowledge and pro-environmental behaviour is one of the most debated outcomes of education in the literature. The return of education is the focus of many studies [11,19,23].

Environmental moral education plays a vital role in countering environmental problems, and the striving goal is to protect and conserve the resources of the planet for a healthy and prosperous life. The effect of EME on PEB has been widely studied across the world. The relationship is diverse depending on the region, religion, culture and many other factors [6,24]. Most of the studies found a positive relationship between EME and PEB; however, still, some studies suggested that a high level of environmental education will not necessarily reflect environmental friendly behaviour [25]. For example, Ek and Soderholm [26] revealed that the relationship between a high level of education (university enrolment) and choice of using green electricity was insignificant. Furthermore, Ayalon et al. [27] found no evidence of education’s effect on recycling behaviour. Wessells et al. [28] found that a high level of education did not persuade consumers to purchase eco-labelled seafood. Finally, Grafton [29] found a negative relationship between water conservation and a high level of education.

Conversely, many studies found out that EME empowers individuals’ understanding of the environment and motivates them to perform pro-environmental behaviours in a range of contexts [30,31]. For example, extant literature is available showing that education promotes recycling behaviour [32,33,34]. Other researchers found that education changed the food choices of individuals, opting to take an environmentally friendly choice. For example, an environmentally educated person usually prefers eco-friendly shopping [35,36]. Berl et al. [37] found out that highly educated people exhibit water-saving behaviour. Similarly, there is evidence from other studies that energy-saving behaviour is also found among educated persons [12,38]. Furthermore, it was found that education is associated with more PEB. For instance, Rowlands et al. [39] found that individuals with awareness of green electricity will emphasise and their raise voices for higher production of eco-friendly electricity. Furthermore, De Silva and Pownall [40] found that to improve environmental quality, college students even opted to sacrifice their financial well-being. A study by Xiao et al. [41] revealed that environmental concerns are higher among those students who are well aware of environmental education. Furthermore, a study by Torgler and García-Valiñas [42] revealed that along with formal environmental education in universities, informal education through print, electronic and social media contributes toward PEB.

Besides the available extant literature on the relationship between EME and PEB, according to Mitchell and Hodson [43], the role of EME can also be considered as an individual resource that provides further support to PEB; the phenomenon follows the conservation of resources (COR) model [44]. The central tenet of the COR model is that people strive to create, protect, maintain and retain resources. Where resources are those objects, individual characteristics, energies or conditions valued by the persons or that serve as a means for the achievement of these objects, individual characteristics, energies or conditions [44]. Examples of resources include self-esteem [45], learned resourcefulness [46], organisational behaviour, behavioural medicine, social work, education and employment [47]. The model also suggests that those with a reliable resource pool are the most “resource secured” and they have developed a good reservoir of resources [48]. Since education is an important and high-quality resource, we believe that it will positively persuade individuals toward conservation of resources through PEB (i.e., gain of resource). The aforementioned discussion suggests that EME will persuade an individual toward PEB; therefore, we propose that environmental moral education is positively related to pro-environmental behaviour.

### 2.2. The Mediating Effect of Psychological Empowerment

The concept of psychological empowerment has been used in organisational and social psychology literature in different research fields such as job enrichment, participative management, alienations and organisational behaviour [49]. There are many inconsistencies among the exact definitions of the psychological empowerment concept, even within the same research area [50,51]. In earlier research work, the term empowerment was used as the factual increase in employees’ power by providing them decision-making authority [52]. However, the term empowerment can be defined as an external or internal process, the internal process of an individual empowered to make effective decisions [53] or simply an act of empowering others [54]. Therefore, the term psychological empowerment has been defined in different contexts in every area of research. In educational research, the term psychological empowerment is the “process of school participants developing the system where they improve their growth and resolve their problems”. According to Conger and Kanungo [55], psychological empowerment is a motivational force which enhances the self-efficiency of organisational members by both formal and informal practices of promoting competence. Moreover, most researchers classified psychological empowerment as a motivational construct facilitating proactive behaviours [56,57]. Empowerment gives the feeling of competency, and empowered individuals believe that they can influence their environment. Empowerment is an active perception of an individual work role rather than a passive one. The antecedents of employee’s psychological empowerment include self-esteem, rewards, control and access to information. The subjective process of empowerment consists of initiating and regulating actions with a positive impact on the job environment. Moreover, managerial effectiveness and innovativeness were obtained due to the fact of increased psychological empowerment [58], thereby leading to improved job performance [59]. The empowering of employees in an organisation influences intrinsic motivation which increases creativity [60]. The mediating effect of psychological empowerment was also studied in earlier research along with other processes such as its mediating role between job satisfaction and psychological climate [56], substantial increases in power and authority on behaviours [61], affective commitment and job satisfaction [62] and the relationship between the transactional leadership and followers’ organisational identification [63].

Recently, consumer empowerment emerged as a promising new research area constituting new concepts about ecological behaviours [64]. Consumer empowerment from social psychology may either refer to the actual power exerted by the consumer or the feeling that consumers observed about the psychological experience of empowerment. The former perspective of consumer empowerment is influenced by the education level of an individual which gives confidence about selecting suitable choices for purchase [65]. Different perspectives have been studied among the relationships of consumers and pro-environmental relationships; however, the role of EME is still not appropriately understood. Consumers’ education plays a vital role in the psychological empowerment of consumers [65]. According to Hayward et al. [66], an individual is empowered when he can resist a hard seller. Similarly, Coppack [67] formulated the National Consumer Council guidelines for the UK that state that consumer education leads to empowerment if more efficient knowledge is transferred to consumers. Furthermore, Mcgregor [65] proposed that the interest of consumers can be protected through education and providing them reliable information so that they have wise and efficient choices. According to Wells and Atherton [68], confident consumers have control and are empowered to have only informed choices because of the education. They further studied the effectiveness of informed citizens at the society level and stated that consumers’ education improves the environment of society by creating informed and active citizens. Besides formal education, informal modes of education and awareness through the internet and print, electronic and social media also empower consumers toward a green ecological environment [65].

Psychological empowerment is a sort of subjective experience of authority and power of an individual. This concept of empowerment is purely a psychological construct; it is based on the individual’s internal decision-making power that allows them to choose products based on available information and their perception of the environment [64]. Empowerment gives a sense of control, and individuals can contribute to improving the sustainability of the environment through environmentally friendly behaviours. Very limited studies have been carried out on the mediating role of psychological empowerment, which is a crucial motivational factor toward sustainable pro-environment behaviour. Thøgersen [69] is among the pioneers who studied the experience of consumer empowerment toward sustainable behaviour and found a significant relationship. Similarly, Mcgregor [65] stated that critical education helps individuals find their inner feeling and power of decision making by empowering them against the status quo and, thus, plays a significant role in sustainable ecological behaviours. Indeed, the feeling of disempowerment among individuals toward environmental problems creates a significant challenge in resolving issues such as climate change [64]. In the current age of globalisation, psychological empowerment becomes particularly relevant as a motivational factor in sustainable consumption. Psychological empowerment gives motivational powers and a feeling is shaped that one’s actions may establish a substantial ancestor of pro-environmental behaviour [56].

The earlier discussion shows that psychological empowerment was used as a mediating factor in several studies examining social, psychological and sustainable environmental variables. We believe that psychological empowerment can be used as a mediating factor between EME and PEB based on the abovementioned literature and the concept of resource caravans proposed by conservation of resource theory [47]. We take a positive resource gain approach toward EME, which is a resource pool capable of orchestrating resource gain in the form of environmentally friendly behaviours [70]. We believe that the EME resource is being utilised on students which creates another resource in the form of motivational psychological empowerment tools which, in turn, results in the conservation of resources through eco-friendly behaviours and sustainable lifestyles. Therefore, psychological empowerment seems to mediate the effect of EME on PEB.

The covariance relationship among three variables was established through a mediation. The variables included an independent variable and an assumed mediating variable and a dependent variable [71]. The shared variance among the independent (EME) and dependent (PEB) variables was investigated through mediation (PEmp). The mediating variable changed the relationship between the independent and dependent variables. Based on the aforementioned discussions, we propose that psychological empowerment mediates the positive relationship between environmental moral education and pro-environmental behaviour.

### 2.3. The Moderating Effect of Islamic Religiosity

The submission of oneself to the instructions of his or her religion is called religiosity. Religiosity gives guidance about living style, which is reflected in the values and attitudes of individuals and societies [72]. These values and attitudes form the behaviours of communities and nationalities. Islam teaches altruism, sustainability and conservation of resources to its followers [21]. Furthermore, those who follow religions in true spirit and have high altruism are found to be actively engaged in sustainable environmental behaviours [73]. Religiosity is always debated as a tool to analyse its effect on consumer behaviour. Religiosity has profound effects on an individual’s lifestyle, thoughts and habits. This is why, for the past few decades, the impact of religiosity on the behaviour of peoples is under immense debate. The importance is increased in the case of a religious country where the majority of the population are followers of the same religion, as in the case of Pakistan, where Islam is their national religion [74].

Religiosity plays a significant role in the green purchasing decision of consumers. Keeping in mind the profound impact of religiosity, several authors have suggested considering the eminence of religiosity in the ecological green environment. Furthermore, it was also observed among the Muslims community that the level of religiosity guides their behaviour to spend moderately and shop environmentally friendly [75]. Moreover, religiosity helps individuals while buying a new product based on the principles of suitability, conservation of resources and environmental concerns such as in the adoption of sustainable clothing consumption [72].

Pro-environmental behaviour leads to safeguarding the natural resources of the environment. Those who possess pro-environmental behaviour are regarded as green consumers in the literature [76]. Evidence is also observed in the earlier literature that religiosity positively reshapes green consumer behaviour that protects the natural ecological cycles of the world. Most of the previous studies developed a measurement scale of religiosity for Christianity, while measuring its impact on the ecological behaviours [77]. However, more recently, Worthington et al. [78] developed a scale for measuring religiosity that can be easily moulded for different religions. Two dimensions of religiosity, interpersonal religiosity and intrapersonal religiosity, were measured in this study. Interpersonal religiosity is personal religious practices, whereas intrapersonal religiosity deals with the matters of the collective society. Furthermore, the relationship between pro-environmental behaviour and Islamic preaching was studied by Mas’od and Chin [79], and a positive and significant linkage was found. According to Islam and Chandrasekaran [19], highly religious Muslim consumers make more efforts to protect the natural environment as compared to less religious consumers. Islamic religiosity introduces the concepts of sustainability and balanced actions, helped by which positive changes in societies can be made by not consuming more than their needs and by assisting the welfare of others [80]. Islam gives the teaching of sustainability, impartiality, balance actions and judicial actions for the protection of the ecological system. In Islam, the human is not the owner of the natural resources of the Earth. Furthermore, Islam gives importance to the protection of natural resources by moderate consumption of resources [75]. This makes religiosity an essential factor toward ecological behaviours because, previously, the moderating role of religiosity has been little studied in this context. Furthermore, the use of religiosity as a moderator in future studies is recommended by Joshanloo and Weijers [81]. Therefore, based on the above discussion, it has been hypothesised that Islamic religiosity will moderate the relationship between EME and PEB. Parallel with these arguments, we believe that Islamic religiosity is a personal resource which, if invested in the resource pool of EME and psychological empowerment, will result in the green ecological behaviours and, therefore, gain additional resources. This assumption is in line with the COR perspective [70]. Giving these arguments, we conceive that students who are provided with EME and have high Islamic religiosity will protect and conserve the environment more efficiently. Thus, we propose that Islamic religiosity plays a moderating role in our study.

## 3. Methodology

### 3.1. Hypotheses

Based on the aforementioned arguments and evidence, we established these hypotheses:

**Hypothesis** **1.** Environmental moral education is positively related to pro-environmental behaviour.

**Hypothesis** **2.** Environmental moral education is positively related to psychological empowerment.

**Hypothesis** **3.** Psychological empowerment is positively related to pro-environmental behaviour.

**Hypothesis** **4.** Psychological empowerment mediates the positive relationship between environmental moral education and pro-environmental behaviour.

**Hypothesis** **5.** The direct positive relationship between environmental moral education and pro-environmental behaviour is expected to be significant for those who are high in Islamic religiosity.

**Hypothesis** **6.** The direct positive relationship between environmental moral education and psychological empowerment is expected to be significant for those who are high in Islamic religiosity.

**Hypothesis** **7.** High Islamic religiosity significantly moderates positive indirect effect of environmental moral education on pro-environmental behaviour, which is mediated by psychological empowerment.

### 3.2. Sampling Procedure

The target population selected for this study were university students because universities produce future leaders, decision-makers and scholars in the political, economic and social sectors [4]. Moreover, the data collected from students were from homogeneous groups where random errors were small. The university students were more concerned about the ecological well-being of nature, as they are studying much literature on these issues in their curriculum [5,6].

Data were collected from several public and private universities of Peshawar (Capital of Khyber Pakhtunkhwa Province, Pakistan). Peshawar is ranked as the 6th largest city in Pakistan, as per the Bureau of Statics of Pakistan, and is currently facing critical ecological issues. Furthermore, very little research work is carried out in the whole country and especially in Peshawar. The survey work was conducted from September 2019 till December 2019. Data were collected from a convenience sample of students from six universities (three public and three private). The public sector universities chosen were University of Peshawar, Agricultural University Peshawar and Islamia College University Peshawar. The private universities visited were Sarhad University of Science and Information Technology Peshawar, City University of Science and Information Technology Peshawar and CECOS University Peshawar. Before starting the collection of data, the heads of the department and class in charge were approached and permission was obtained. A brief session was conducted in the classrooms to the prospective students about the aims and nature of the research survey. Moreover, the questionnaire survey also included a cover letter stating that the aim of this study was for research purposes only and that the respondents would be kept anonymous and confidential. Students were also informed that their participation in the survey was voluntary and that they could withdraw their participation at any stage of data collection. Students were given enough time to respond and complete a pen and pencil survey questionnaire and return it to the researchers anonymously in envelopes. A total of 500 surveys were distributed out of which 461 were returned with a yielding response rate of 93%. Of the returned questionnaires, 32 were found to be invalid due to the fact of incompleteness or careless responses, hence, making available 429 usable questionnaires [82]. The sample consisted of 281 (65%) male respondents and 148 (34.4%) female respondents. Among the valid respondents, 77.8% were undergraduate students, whereas 22.2% were postgraduate students.

### 3.3. Instruments

The measures for this study were adapted from previous literature and it was modified slightly as per the demands of the current study. The variables were evaluated using a five-point Likert scale ranging from 1 for “strongly disagree” to 5 for “strongly agree”. The scale for environmental moral education consisted of two parts having eight items—formal and informal education. The former scale was adopted from the Pérez-Rodríguez et al. [83] study, whereas the was adopted from the Varela-Candamio et al. [84] study. The psychological empowerment scale was measured with five items adapted from Spreitzer’s [85] and Van Kleef et al. [86], which were already being tested by Hartmann et al. [87] under the specific case of psychological empowerment related to pro-environmental consumer behaviour. The scale of Islamic religiosity was adapted from the ten item Religious Commitment Inventory scale devised by Worthington et al. [78]. This scale is superior to older scales because it was developed carefully for followers of different religions rather than just a single religion. This is why it is more easily adapted and considered better than other scales. Pro-environmental behaviour was assessed by a 13 item scale adapted from Kaiser et al. [88]. The Cronbach’s (α) were measured for all the scales which were within the acceptable range as shown in Appendix A along with the title of each variable. Gender and age are the most influencing variables when the target population is university students [89]. The literature shows that these variables have a profound influence on the green behaviour of students; therefore, in this study, we examined the impact of these factors as control variables. All scale items for this study are presented in Appendix A.

### 3.4. Analytical Approach and Construct Validity

The potential for common method variance (CMV) was a concern because data were obtained from individual participants in a cross-sectional study [90]. To assess CMV, Harman’s one-factor test was carried out [91]. In this test, all the principal constructs are entered into a principal component factor analysis. Evidence of CMV exists when a single factor appears from the analysis or when one general factor accounts for the majority of the covariance in the interdependent and dependent variables. The results showed that five factors based on eigenvalue in excess of 1, accounted for 64.4% of variance, while the highest single factor, representing EME, accounted for 25.9% of the variance. This indicates that CMV did not appear to be a substantial issue in the data of this study.

The proposed model was examined via SPSS and AMOS versions 23 using a two-step analytical procedure recommended by Anderson and Gerbing [92]. That is, the model variables were analysed first through confirmatory factor analysis (CFA) using maximum likelihood estimation to evaluate the distinctness of the main study constructs before conducting the second step which was structural equation modelling (SEM) [93]. These approaches are powerful statistical tools for testing association among latent constructs and examine a priori hypotheses regarding relationships between observed and latent variables [94].

## 4. Results

### 4.1. Measurement Model Evaluation

The CFA results provided initial evidence of construct validity, as each item was fit to its latent factor (e.g., all psychological empowerment items created a psychological empowerment factor). Moreover, the goodness-of-fit of the tested model was assessed using four established model fit indices including Chi-square (χ^2^), the root mean square error of approximation (RMSEA), the Tucker–Lewis index (TLI) and comparative fit index (CFI) [93,95]. When χ^2^ is significant, CFI and TLI are greater than 0.9 and RMSEA is less than or equal to 0.08, the model fit is satisfactory [93]. After allowing residuals to correlate [96], the expected four-factor measurement model displayed adequate fit across the sample: Chi-square = 1134.60, CFI = 0.949, TLI = 0.945, RMSEA = 0.048, SRMR = 0.034. These adequate indices provided robust support for the validity of our study.

To establish the validity and reliability of constructs, individual item reliability, composite reliability, convergent validity and discriminant validity were evaluated [97]. All of these coefficients exceeded the recommended thresholds (i.e., Cronbach’s α > 0.60, composite reliability > 0.70), thus accepting the reliability of the measures used [98]. To ascertain the convergent validity, the average variance extracted (AVE) for each latent factor was analysed. In general, the AVE values higher than the required minimum of 0.5 provide support for convergent validity [99]. As shown in Table 1, the AVE values were higher than the recommended threshold, hence, indicating satisfactory convergent validity. For valid discriminant of a construct, the square root of each construct’s AVE should be larger than its correlations with other constructs [99,100]. The results depicted in Table 1 fulfilled the said criterion confirming that the measurement model has the required discriminant validity.

### 4.2. Structural Equation Model Path Analysis

The fit statistics of the SEM model were Chi-square = 772.1, RMSEA = 0.049, CFI = 0.950 and TLI = 0.944, implying that the model had a good fit. Analytical results of the study revealed that the students’ environmental moral education had a significant influence on their pro-environmental behaviour (β = 0.11, *p* < 0.05), providing support to **H1**. Moreover, the results of the study showed that students’ environmental moral education had a significant effect on their psychological empowerment (β = 0.18, *p* < 0.01), and their psychological empowerment was further found to significantly influence pro-environmental behaviour (β = 0.13, *p* < 0.05), leading us to accept **H2** and **H3**. The significance of both direct and indirect paths indicated partial mediation. In addition, age (β = 0.09, *p* > 0.05) was found to be insignificant, while gender (β = 0.12, *p* < 0.05) was significant. However, it is noteworthy that neither the insignificance of age nor significance of gender deteriorated the results of our main model. The results depicted in Table 2 show the structural equation model path analysis outcomes.

### 4.3. Mediating Effect of Psychological Empowerment

**Figure 2:**
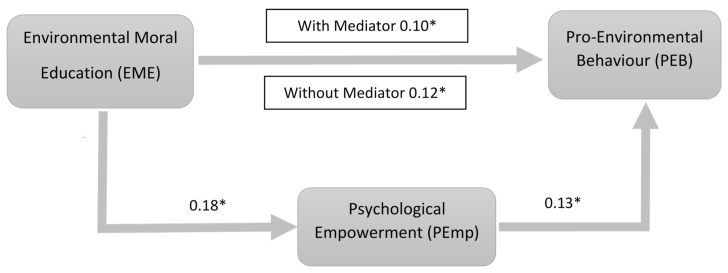
Sobel’s test result of partial mediation. Coefficients both in the presence and absence of mediator *p* < 0.05. Where * shows significance (*p* < 0.05).

The PROCESS macro v.3.0 for SPSS (Model 4) was used to examine the mediating role of psychological empowerment. The results show that environmental moral education was positively related to psychological empowerment (β = 0.18, *p* < 0.01, SE = 0.05, 95% CI = [0.07, 0.28]), and psychological empowerment was positively related to pro-environmental behaviour (β = 0.13, *p* < 0.01, SE = 0.04, 95% CI = [0.06, 0.22]). The residual direct effect was also found significant (β = 0.06, *p* < 0.05, SE = 0.04, 95% CI [L = 0.01, U = 0.14]). Therefore, psychological empowerment played a partial mediating role in the effect of environmental moral education on pro-environmental behaviour (indirect effect = 0.02, SE = 0.01, 95% CI [L = 0.010, U = 0.047]), supporting **H4**. We further conducted Sobel’s test and found similar results as shown in Figure 2. Psychological empowerment significantly partially mediated the relationship between environmental moral education and pro-environment behaviour (Sobel Z = 2.55, *p* < 0.05). That is, environmental moral education had a significant impact on pro-environmental behaviour indirectly through psychological empowerment.

### 4.4. Moderated Mediation

**Figure 3:**
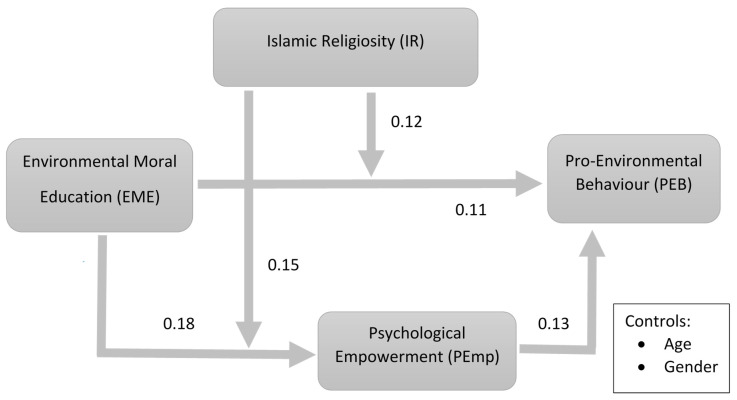
Research model.

**Figure 4:**
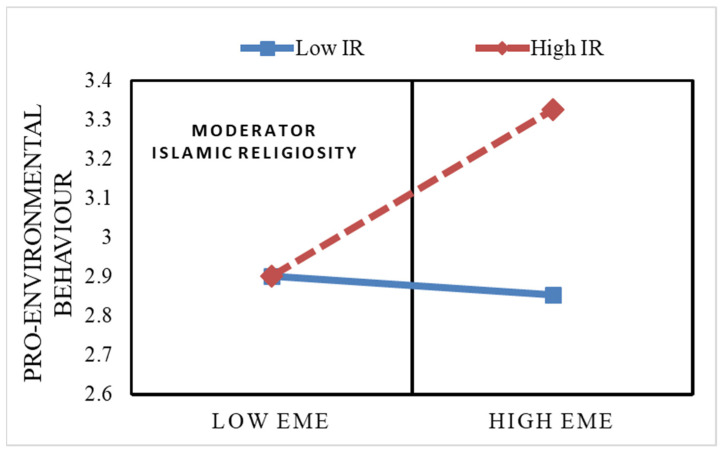
Pro-environmental behaviour as a function of environmental moral education and Islamic religiosity.

In hypotheses **H5**, **H6**, and **H7**, the current study expected that Islamic religiosity would moderate the direct and indirect effects of environmental moral education on pro-environmental behaviour via psychological empowerment. We examined the moderated mediation hypotheses (Figure 1) with PROCESS macro v.3.0 (Model 8), and the results are provided in Figure 3. The results show a significant positive interaction of environmental moral education and Islamic religiosity on pro-environmental behaviour. This indicates that Islamic religiosity strengthened (positively moderated) the effect of environmental moral education on pro-environmental behaviour (β = 0.12, 95% CI [L = 0.04, U = 0.21], *p* = 0.004) thus leading us to accept **H5**. Similarly, the significant positive interaction of environmental moral education and Islamic religiosity on psychological empowerment also showed that Islamic religiosity increased the strength of the link between environmental moral education and psychological empowerment (β = 0.15, 95% CI [L = 0.04, U = 0.27], *p* = 0.007) therefore supporting **H6**. As depicted in Figure 4, the link between environmental moral education and pro-environmental behaviour became stronger with an increase in Islamic religiosity. Moreover, it was also found that EME’s, PEmp’s and PEB’s relationships depended on students’ Islamic religiosity. The increase in Islamic religiosity strengthened the positive association between environmental moral education and psychological empowerment which increased the indirect effect of environmental moral education on pro-environmental behaviour through psychological empowerment. Both of them increased with an increase in Islamic religiosity (for the former, if Islamic religiosity = −SD, b = 0.03, 95% CI [L = −0.014, U = 0.057], if Islamic religiosity = 0, b = 0.080, 95% CI [L = 0.0009, U = 0.160], if Islamic religiosity = +SD, b = 0.019, 95% CI [L = 0.083, U = 0.311]; whereas for the latter indirect effect of EME on PEB, if Islamic religiosity = –SD, b = 0.005, 95% CI [L = −0.006, U = 0.025], if Islamic religiosity= 0, b = 0.019, 95% CI [L = 0.005, U = 0.043], if Islamic religiosity = +SD, b = 0.034, 95% CI [L = 0.089, U= 0.072]). Thus, the proposed hypotheses were supported by the results of the study.

## 5. Discussions

The current research was carried out to find the relationship between the environmental moral education of university students and the tendency of students toward pro-environmental behaviour with the mediating effect of psychological empowerment and the moderating effect of Islamic religiosity. The results show that environmental moral education was directly and positively associated with pro-environmental behaviour. Furthermore, the study proved that psychological empowerment partially mediated the positive relationship between environmental moral education and pro-environmental behaviour. In addition to this, it was also proved that Islamic religiosity played a positive moderating role. The environmentally educated students with a high level of Islamic religiosity were found to behave more environmentally friendly as compared with those having lower Islamic religiosity. Similarly, the direct relationship between environmental moral education and pro-environmental behaviour was significant among students with high psychological empowerment and vice versa.

This study plays a vital role in understanding the contributing role of environmental moral education in a promoting pro-environmental or ecological lifestyle. There is very little research work on the effect of environmental moral education on ecological behaviour mediated by psychological empowerment. Our results show that environmental moral education motivates students toward green lifestyles both directly and indirectly through the mediation effect of psychological empowerment. This finding supports previous claims that environmental education fosters the relationship of students with nature and that they are motivated toward green lifestyles [20,101]. Similarly, Otto and Pensini [102] found that nature-based environmental education is a promising approach to increase pro-environmental behaviour. Among others, Ballantyne and Packer [103] found that students find natural environmental protection learning an exciting subject and this changes their attitudes toward environment, desires and behaviours. It has been shown that environmental moral education positively affects pro-environmental behaviour; therefore, educational institutes can incorporate more environmental educational learning materials for promoting ecological behaviour. In addition to fostering environmental education, Islamic religiosity provides guidelines to behave ecologically and conserve natural resources, thereby offering an enduring approach to green behaviour. Overall, environmental moral education is a very efficient way of promoting green ecological lifestyles, because it empowers students about their actions regarding nature.

The present study pointed out that psychological empowerment partially mediates the relationship between environmental moral education and pro-environmental behaviour; therefore, empowering students to protect and conserve nature and behave environmentally friendly is important. The concept of psychological empowerment gives the perception of authorisation. Individuals with high psychological empowerment perceptions think that they have a greater role of self-responsibility in protecting nature. Therefore, they will be more willing to perform pro-environmental behaviours. This is an important contribution of this study, because previous studies have mostly considered the role of attitudes and affection as mediators between environmental moral education and pro-environmental behaviour [5,89]. While drawing on the conservation of resource theory, this study suggests that environmental moral education promotes psychological empowerment as a resource gain among the students which promotes their pro-environmental behaviour. Therefore, contributing to the theoretical development of the literature by showing that psychological empowerment can be integrated into COR theory as an antecedent of pro-environmental behaviour. This finding is also in line with the earlier research claims that, presumably, psychological empowerment is a motivational factor toward the green consumption process [64,104]. The study further proved that psychological empowerment motivates pro-environmental behaviour. These findings imply that psychological empowerment gives the power of control over environment-related issues that concern individuals and motivates them toward a greener lifestyle, for example, by adopting water and electricity savings. Overall, the outcome of this study shows that the psychological empowerment of students is a useful motivational tool that will cause green ecological behaviours.

Furthermore, the moderating impact of Islamic religiosity on pro-environmental behaviour was investigated in this study. It was found that Islamic religiosity moderated the relationship between independent and dependent variables. The results show that religiosity influenced the ecological behaviour of Muslims. The following of religiosity is highly personal in nature and, therefore, the behaviour adaption as per the religious instructions also depends on the individual’s religious commitment. The same was also proved through the finding of this study, that individuals with a high degree of religious commitment are more environmentally friendly and vice versa. This is another significant contribution of the study, as it extends the role of religiosity into pro-environmental behaviour. The study found that students’ Islamic beliefs about the environment reinforce the role of environmental moral education in promoting pro-environmental behaviour. The earlier literature is also in line with our findings. For example, Alam et al. [75] found that Muslims consume moderately due to the instructions on the conservation of resources. It was also reported that religious consumers are less greedy and selfish and are more altruistic [105]; therefore, they are involved in sustainable and better environmental deeds. Similar findings were also reported by Mohammad and Som [106]; according to their investigation, religiosity has a vital role in developing sustainable green behaviours in consumers. Among others, Rice [21] believes that religiosity has, nonetheless, an effect on environmental consumers’ behaviour. The results of the current study confirmed a positive moderating role of Islamic religiosity on green ecological lifestyles. However, it is worth noticing that religiosity is highly personal in nature and one person’s religiosity is quite different from another [107].

## 6. Limitations and Future Directions

The current study has a few limitations which can be addressed as avenues for future studies. The scope of our study was very limited in terms of the targeted population of society which can be prolonged by incorporating working professionals, housewives and adults from different walks of life. The current study focused only on university students, which is a very limited proportion of the population; furthermore, their behaviour might be changed in practical life depending upon the lifestyle of ordinary individuals. In addition, the green ecological behaviour pattern of peoples in different stages of life and professions can be compared in the future and very good results can be obtained. The findings of the current study can be compared with the results of other parts of the world with different religions and cultures. This will help in understanding the cultural and behavioural differences across the world toward green ecological behaviours.

Among other limitations of the current study was that the model of the study was tested under Islamic religiosity for Muslims; therefore, the model can be applied to a section of the society only. Furthermore, the results obtained in the current study are based on cross-sectional data resulting in harder causal inferences. It might be more useful if future scholars examined the interplay among the study variables with a longitudinal design. The current study lacks the experimental research approach which is required for measuring psychological empowerment [108]. The theory of COR can be extended for the current study; however, further studies are required to test the COR theory on the psychological empowerment-based reinforcement process in green ecological lifestyle by integrating additional antecedents into the proposed theoretical model.

## 7. Conclusions

In conclusion, this study confirms the significant role of environmental education, which contains both formal and informal education in fostering students’ pro-environmental behaviours. Environmental moral education gives the feeling of psychological empowerment in the presence of Islamic religiosity code of conduct which also restrains their followers from damaging the environment. The findings of the current research work are also in line with earlier studies [9,21,42,67], that is, environmental education empowers students toward green behaviour. This study revealed that students were psychologically empowered owing to a high level of knowledge due to the fact of environmental moral education and, therefore, had a high tendency to engage in environmentally friendly behaviours. Environmental education empowers students toward green behaviours. Furthermore, Islamic religiosity also plays a positive role in improving the pro-environmental behaviours of students. Students with high religiosity were found to be engaged in greener lifestyles than lower religiosity ones. Therefore, it is suggested that both formal and informal environmental moral education and religious preaching must be promoted in higher institutions for green and sustainable behaviour outcomes. This conclusion is also supported by earlier research work which states that environmental education results in pro-environmental behaviours [5,6]. Moreover, environmental education must focus not only on the sharing of the information provided but should also foster ecocentrism and a wish to conserve nature in education.

## Figures and Tables

**Table 1:** Descriptive statistics and correlation matrix.

Variable	CR	AVE	M	SD	EME	PEB	IR	PEmp
EME	0.93	0.64	2.8	0.94	(0.80)			
PEB	0.95	0.62	2.6	0.73	0.119 *	(0.78)		
IR	0.95	0.67	3.8	0.90	−0.080	0.184 **	(0.82)	
PEmp	0.90	0.64	2.7	0.94	0.182 **	0.203 **	0.184 **	(0.80)

Variances extracted are on the diagonal; correlations are off-diagonal. * *p* < 0.05; ** *p* < 0.01, AVE: average variance extracted, CR: composite reliability, EME: environmental moral education, IR: Islamic religiosity, M: mean, PEB: pro-environmental behaviour, PEmp: psychological empowerment.

**Table 2:** Structural equation model path analysis results.

Path		Coefficient	SE	*t*-Value
*Controls*				
Pro-Environmental Behaviour	← Age	0.09 *	0.07	1.83
Pro-Environmental Behaviour	← Gender	0.12 *	0.07	2.46
*Main effects*				
Pro-Environmental Behaviour Environmental Moral Education	←	0.11 *	0.04	2.08
Psychological Empowerment Environmental Moral Education	←	0.18 **	0.05	3.18
Pro-Environmental Behaviour	← EME_X_IR	0.15 **	0.04	2.90
Psychological Empowerment	← EME_X_IR	0.12 **	0.05	2.41

Notes: * *p* < 0.05; ** *p* < 0.01; EME_X_IR: interaction term of environmental moral education and Islamic religiosity. Whereas arrow shows the direction of relationship.

## Data Availability

The data presented in this study are available on request from the corresponding author. The data are not publicly available due to privacy.

## Appendix A

**Demographics questions**

1. Gender _____________________
2. Age _____________________
3. Level of education _____________________
4. You are specialised in (Major) _____________________
5. Have you ever attended any course, seminar or lecture related to environmental education when you were a student?

a. Yes b. No

**Environmental Moral Education (α = 0.93)**

1. Facing the current environmental problems, it is a priority to integrate environmental education at university.
2. Environmental education should especially work on the development of skills such as critical thinking, reflexive decision-making and participation.
3. For environmental education to be as effective as possible, there should be a commitment from the entire educational community.
4. I believe that including environmental education at university can contribute to changing the environmental behaviour of the whole community.
5. I think it is important that all teachers receive environmental training.
6. Media (watch TV and/or read newspapers) improve my knowledge about environmental issues.
7. I do use the Internet to improve my knowledge about environmental issues.
8. My social interactions (with family, friends, etc.) improve my knowledge about environmental issues.

**Psychological empowerment (α = 0.90)**

1. Doing something about climate change makes me feel that I’m making a difference.
2. Reducing my personal carbon emissions gives me a feeling of power, because my choice as a consumer counts.
3. Participating in the reduction of climate change makes me feel I can have an impact on what happens.
4. Taking action against climate change makes me feel I have the power to change things.
5. Switching to green electricity I feel more powerful because I vote with my purchasing decisions.

**Islamic Religiosity (α = 0.95)**

1. I actively participate in the activities of my local religious institute (mosque of your colony) or privately arranged religious activities (e.g., Milad).
2. I enjoy more spending time with people of my religion as compared with people of other religions.
3. I keep well informed about my local religious group and have some influence on its decisions.
4. I regularly make financial contributions to my religious organisation (e.g., mosques).
5. My religious beliefs lie behind my whole approach to life.
6. Often spend time trying to increase understanding of my faith.
7. It is very important for me to spend periods of time in private religious thought and reflection.
8. My religious beliefs influence all my dealings in life.
9. My religion is especially important to me because it answers many questions about the meaning of life.
10. I often read books and magazines about my faith.

**Pro-Environmental Behaviour (α = 0.95)**

1. I buy products in refillable packages.
2. I buy products with green labels.
3. I buy products that come with minimal packaging.
4. I buy paper and plastic products that are made from recycled materials.
5. I avoid buying products that have potentially harmful environmental effects.
6. I limit the extra use of water.
7. I use energy-efficient household devices.
8. I reduce the consumption of paper in daily use.
9. I use recycled paper.
10. I put empty bottles and cans in a recycling bin.
11. I sort my household trash for recycling.
12. I recycle used plastic.
13. I boycott companies known to harm the environment.
14. I will sign a petition in support of promoting the environment.
